# Exploring the anticancer potential of green silver Nanoparticles–Paclitaxel nanocarrier on MCF-7 breast Cancer cells: an in vitro approach

**DOI:** 10.1038/s41598-025-06275-4

**Published:** 2025-06-20

**Authors:** M. Bassam Aboul-Nasr, Alaa A. Yasien, Sabah S. Mohamed, Yahya Bassam Aboul-Nasr, Marwa Obiedallah

**Affiliations:** 1 Botany and Microbiology Department, Faculty of Science, Sohag University, Sohag, 82524 Egypt; 2 Health office, military strategic center, Ministry of Health, New Cairo, Egypt

**Keywords:** Paclitaxel, Nanocarrier, Silver nanoparticles, *A. fumigatiaffinis*, Anticancer activity$\circ_{\lambda}$, Biotechnology, Microbiology, Oncology

## Abstract

Paclitaxel (PTX) is a potent chemotherapeutic agent limited by poor solubility and adverse effects. To address these challenges, we developed a novel nanocomposite combining PTX purified from the endophytic fungus *A. fumigatiaffinis* PP235788.1 with biosynthesized silver nanoparticles (AgNPs) from the same fungal strain. The PTX-AgNP conjugate (28.48 ± 0.13 nm) was characterized by UV-Vis, XRD, and TEM, revealing monodisperse AgNPs (14.50 ± 0.58 nm) as the core component. In vitro studies demonstrated significant cytotoxicity against MCF-7 breast cancer cells (IC₅₀= 1.7 µg/mL, *p* < 0.001), with 5-10-fold greater efficacy than AgNPs alone. Annexin V/PI staining and DNA fragmentation assays confirmed apoptosis induction, highlighting the conjugate’s enhanced anticancer activity. This eco-friendly nano platform synergizes PTX’s therapeutic effects with AgNP-mediated targeting, offering a promising strategy to reduce side effects while improving tumor-specific cytotoxicity for advanced cancer therapy.

**Supplementary Information:**

The online version contains supplementary material available at 10.1038/s41598-025-06275-4.

## Introduction

Today, chemotherapy remains the predominant modality in oncological treatment. To ensure effective intervention, it is imperative to administer an intracellular dosage of chemotherapeutic agents with precision and adequacy^1^. Nevertheless, in the context of chemotherapy, a critical equilibrium exists between therapeutic response and toxicity; subtherapeutic dosing may render the drug ineffective, whereas excessive dosing may precipitate systemic toxicity^2^. Among chemotherapeutics, paclitaxel (PTX) stands out for its broad activity against breast, lung, and ovarian cancers and ranks among the most potent chemotherapeutic agents. Its anticancer activity enhances microtubule stability while obstructing cell cycle progression at the G2/M transition, ultimately triggering apoptosis^3^. Paclitaxel is characterized by a narrow therapeutic index, with significant obstacles to clinical efficacy arising from its low aqueous solubility, limited permeability, and pronounced adverse effects. Numerous investigations have been undertaken to explore various formulations of PTX, expecting that these may enhance therapeutic effectiveness^4^. Recently, nanoparticle-based drug delivery systems have been introduced, providing significant advantages in enhancing accumulation and retention within tumors through the enhanced permeability effect while potentially reducing the adverse effects of cancer treatments^5^. These systems, characterized by sub-40 nm particle sizes, exhibit improved efficiency in passive tumor targeting and act as potential carriers to enhance PTX’s therapeutic efficacy while minimizing adverse effects.

Nanocarriers can prolong the half-life of therapeutic agents in clinical treatments, demonstrate enhanced pharmacokinetic characteristics, and promote increased patient adherence. PTX has been explored within various advanced nanodrug delivery mechanisms in this context. These platforms include polymer-based nanoparticles, lipid formulations, polymer-linked conjugates, inorganic nanomaterials, carbon nanotube structures, nanocrystalline compounds, and cyclodextrin-derived nanoparticles^6^.

The nanoparticles encapsulating PTX exhibit variability in their physicochemical attributes, leading to distinct cellular processes across different cell types^7^. Recent studies have encapsulated PTX within halloysite, a clay-based material, and coated it with a pH-sensitive polymer, enabling controlled drug release and enhancing its therapeutic efficacy^8^. Additionally, another study on paclitaxel applied an ultra-thin oppositely charged polymer coating on nanoparticles with limited solubility to improve their dispersion stability. The release kinetics of PTX from these nanocapsules were effectively regulated within 10–20 h^9^.

Among the various metal nanoparticles, silver nanoparticles (AgNPs) remain of considerable interest in biomedicine due to their size-dependent photophysical characteristics, thereby being regarded as one of the most efficacious nanoparticles^10,11^. Consequently, research has been aimed at elucidating the possible applications of AgNPs in cancer diagnostics and therapy remains ongoing. AgNPs are recognized for their diverse therapeutic properties, which include bioactive properties, antimicrobial antiviral properties, modulating inflammatory responses, and inhibiting angiogenesis^7,12^. Various physical, chemical, and biological approaches are utilized for AgNP synthesis, offering benefits such as biocompatibility, low toxicity, environmental sustainability, and selective safety for healthy cells^13,14^.

Silver nanoparticles may serve as promising candidates for combination therapies, as recent findings indicate that they influence the activity of permeability glycoprotein (Pgp), thereby enhancing the effectiveness of treatment against resistant cancer cell lines. Moreover, AgNPs induce DNA strand disruptions and genomic instability, leading to apoptosis and contributing to their genotoxic properties^15,16^.

Following our study, the biosynthesized silver nanoparticles incorporating paclitaxel using the endophytic fungus *A. fumigatiaffinis*, which was used for the first time as a paclitaxel and silver nanoparticles producer, were identified as the optimal medium for targeting neoplastic cells. Consequently, we employed silver nanoparticles, which exhibit biocatalytic and photocatalytic properties, are facile to synthesize, and are economically viable as pharmaceutical carriers in our analysis. We aimed to evaluate their anticancer effects on the MCF-7 cell line.

## Methods

### Plant material collection

Specimens of *A*. *judaica* were collected from Wadi Abu Shih, Red Sea Governorate, Egypt (33°20’–33°30’E, 26°30’–26°44’N) during March–April 2022. No permits were required for collection as Egyptian regulations exempt non-endangered desert flora. Voucher specimens (SHG-AJ01) were deposited at Sohag University Herbarium (institutional acronym: SHG) and identified by Dr. Ahmed Elkordy, Associate Professor of Plant Taxonomy, Botany Department, Faculty of Science, Sohag University. Specimens are publicly accessible for verification.

### Fungal culture and Paclitaxel extraction

The endophytic fungus *A. fumigatiaffinis* PP235788.1, isolated from *Artemisia judiaca* plants, was selected for this study based on its high Taxol production yield, as demonstrated in previous research^17^. The fungal strain was maintained on dextrose-enriched potato agar medium (PDA), which was prepared by dissolving 250 g of potato extract, 20 g of dextrose, and 18 g of agar in 1 L of distilled water.

For Taxol production, a fresh culture of *Aspergillus fumigatiaffinis* was inoculated into potato dextrose broth (PDB) using a 1% suspension containing 2 × 10⁸ spores per milliliter, derived from six-day-old pure cultures. The inoculated PDB (50 mL in 250 mL Erlenmeyer flasks) was incubated under optimal conditions. Following incubation, the fungal mycelia were removed from the liquid medium using Whatman No. 1 filter paper. The filtrate was then subjected to paclitaxel extraction with an equal volume of chloroform and analyzed via thin-layer chromatography (TLC), as previously reported^17^.

### Purification of Paclitaxel

Paclitaxel was purified utilizing the Puriflash 4100, an automated flash chromatography system (Interchim, Montluçon, France), employing a semipreparative technique and equipped with a system comprised of a high-performance liquid chromatography (HPLC) unit joined to a quaternary pump, a photodiode array (PDA) detector covering 190–840 nm, a fraction collector, and a sample injection module. Interchim software version 5.0 (Montluçon, France) was employed for operation and data management. Two grams of the crude extract were dissolved in methanol (50 mL) and mixed with a minimal quantity of silica as the stationary phase. Fractionation was accomplished using automated flash chromatography with pre-filled silica columns (INTERCHIM PF-30SI-HP), employing a methanol-chloroform gradient for elution at a flow rate of 15 mL/min and a pressure of 22 bar.

### Biosynthesis and purification of silver nanoparticles (AgNPs)

Silver nanoparticles (AgNPs) were biosynthesized using a paclitaxel-producing fungal filtrate (*A. fumigatiaffinis* PP235788.1); after fungal growth on PDB for 8 days at 28 ± 1.0 ℃. Fungal mycelia (10 g wet weight) were thoroughly washed several times with deionized water, then soaked in 100 mL deionized water for 3 days. Eventually, the obtained fungal filtrate was adjusted at pH 8 and combined with 2 mmol L⁻¹ silver nitrate. The process was conducted at 60 °C over 24 h. Following biosynthesis, the AgNPs were purified by high-speed separation at 10,000 rpm for 10 min at − 4 °C. The particles were washed three times by centrifugation and re-dispersion in deionized water to remove residual unconverted silver ions. Subsequently, the AgNPs were washed twice with ethanol, centrifuged again, and dried at 60 °C.

### Preparation of agnps@ptx nanocarrier

To synthesize the AgNPs@PTX nanocarrier, 25 mg of purified AgNPs were dispersed in 10 mL of a 1:1 (v/v) deionized water-glycerol mixture. Separately, 10 mg of purified paclitaxel (PTX) was dissolved in 10 mL of glycerol. The PTX solution was combined with the AgNPs suspension (1:1 v/v) and mixed using a magnetic stirrer in a dark environment for 30 min. The reaction mixture was then kept at 25 °C for 1 day to facilitate the interaction between PTX and AgNPs. Post-incubation, the solution was sonicated and, after that, subjected to high-speed separation at 12,000 rpm for 30 min at − 4 °C, after which the supernatant was collected and dialyzed against deionized water for 12 h to remove unbound PTX and other impurities. The final AgNPs@PTX nanocarrier suspension was stored at − 20 °C until further use^18^. The synthesized nanocarriers were subsequently characterized.

### Characterization

The UV-visible spectra of AgNPs and AgNPs@PTX nanocarrier were analyzed in the 300 to 600 nm wavelength range via a UV-Vis spectrophotometer (JENWAY 7315, UK). Each measurement was repeated three times, and the mean optical density values were considered for analysis.

Transmission electron microscopy (TEM) imaging was conducted at the Electron Microscopy Unit, University of Assiut, using a JEM100CX11 microscope operated at 100 kV with a resolution of 0.23 nm. For sample preparation, 7 µL of AgNPs or AgNPs@PTX nanocarrier suspension was loaded on carbon film-coated grids and left to dry at ambient temperature for 24 h. The size distribution of nanoparticles was determined by measuring 60 individual particles, and the data were statistically analyzed using the Gaussian model. A histogram of nanoparticle size distribution was generated using Origin software (Version 2025, OriginLab Corporation, Northampton, MA, USA).

The structural properties of AgNPs and AgNPs@PTX nanocarrier nanocarrier were examined using X-ray diffraction (XRD) analysis performed using a Bruker D8 Advance diffractometer. Data acquisition was carried out over a 2θ range of 10° to 80° with a step size of 0.2°.

The surface charge of AgNPs was evaluated using a Zetasizer Nano ZN instrument (Malvern Panalytical Ltd., United Kingdom) via electrophoretic light scattering (ELS). Samples were loaded into folded capillary cells (DTS1070, Malvern), and the Smoluchowski model was applied to calculate zeta potential from electrophoretic mobility. Three independent measurements were recorded per sample, with results reported as mean ± standard deviation.

Organic functional groups in the fungal extra-enzymes and the AgNPs were analyzed using an ALPHA II spectrometer (Bruker) in attenuated total reflection (ATR) mode. Spectra were acquired over 4000–500 cm⁻¹ (resolution: 4 cm⁻¹, 32 scans per sample), with background subtraction (ambient air). Data was processed via OPUS 7.2, Bruker (software) to identify peak assignments.

### Cell viability (MTT Assay)

Cell survival was assessed through the MTT dye reduction assay (3-(4,5-dimethylthiazol-2-yl)−2,5-diphenyltetrazolium bromide), following the method outlined by^19^. In summary, MCF-7 breast cancer cells were plated in flat-bottom 96-well microplates (Corning^®^ 96-well NBS™ Microplate, Corning Inc., Corning, NY, USA) with a cell count of 2 × 10⁵ cells/well and kept for 24 h at 37 °C in a moisture-regulated environment with 5% CO₂ to facilitate cell adhesion.

A series of dilutions for AgNPs (0.1–100 µg/mL) and AgNPs@PTX (0.01–50 µg/mL) were prepared, each tested in triplicate. Additionally, six vehicle control wells containing either culture medium alone or 0.5% DMSO were included in each microplate. Following a 24-hour incubation, the culture medium was replaced with 100 µL of phenol red-free RPMI-1640 medium, and 10 µL of a 12 mM MTT stock solution was introduced into each well. The plates were maintained at 37 °C with 5% CO₂ for 4 h to allow formazan crystal formation.

After incubation, 85 µL of the medium was gently removed from each well, followed by adding 50 µL of DMSO to dissolve the formazan crystals. The plates were mixed carefully and incubated at 37 °C for 10 min to achieve complete dissolution. Optical density (OD) readings were taken at 570 nm using a plate reader (SunRise, TECAN, Inc., USA).

The cell survival percentage was determined by applying the following equation:

$$\:\text{C}\text{e}\text{l}\text{l}\:\text{v}\text{i}\text{a}\text{b}\text{i}\text{l}\text{i}\text{t}\text{y}\:\left({\%}\right)=\left(\frac{\text{O}\text{D}_{1}}{\text{O}\text{D}_{2}}\right)\times\:100$$

Where OD₁ denotes the average optical density of the treated cells, while OD₂ corresponds to the average optical density of the control cells, the relationship between cell viability and drug concentration was plotted to generate survival curves. The compound’s concentration needed to reduce cell viability by 50% (IC₅₀) was calculated using GraphPad Prism software (Version 10.3.0, San Diego, CA, USA).

### Cell apoptosis assay

Cellular DNA content across cell cycle phases was analyzed using propidium iodide (PI) staining, as described by^20^. Briefly, MCF-7 human breast adenocarcinoma cells were exposed to the IC₅₀ concentration (1.7 µg/mL for AgNPs@PTX; 15 µg/mL for bare AgNPs), collected by centrifugation, rinsed with PBS, fixed in 70% cold ethanol, and maintained at 4 °C for 24 h. After fixation, the cells were incubated with RNase A at 100 µg/mL. To remove RNA and stain with PI (50 µg/mL) for DNA content analysis. The samples were labeled with FITC-tagged Annexin-V to identify apoptotic cells according to the supplier’s protocol. Flow cytometric analysis using a Becton-Dickinson FACScan (San Jose, CA, USA) was used to analyze cell population dynamics across the cell cycle’s G₁, S, and G₂/M phases.

### Histological staining and apoptosis evaluation

For histopathological evaluation, 50 µL of AgNPs or AgNPs@PTX (at IC₅₀) was applied per slide (3 slides/group). The slides were air-dried and fixed in 100% methanol for 10 min. Rehydration was performed through a graded ethanol series (100%, 90%, 75%, and 50% ethanol), followed by a rinse in distilled water for five minutes.

The slides were stained with filtered hematoxylin for three minutes and washed twice with distilled water. Counterstaining was performed using filtered eosin for five seconds, followed by another rinse in distilled water. The stained slides were progressively dehydrated using an increasing ethanol gradient, cleared with xylene, and mounted with Canada balsam beneath coverslips.

Ten randomly selected microscopic fields per slide were analyzed under a light microscope at 400× magnification, and images were acquired using a Canon digital camera mounted on the microscope. Fields were selected based on the presence of apoptotic cells. The photomicrographs were examined qualitatively for apoptotic morphological characteristics, such as cell shrinkage, chromatin condensation, and membrane blebbing, following the methodology outlined by^21^.

### Statistical analysis

All experiments were conducted in triplicate. The data were assessed through one-way and two-way ANOVA, followed by Tukey’s post-hoc test for multiple comparisons. Statistical evaluations were performed using Origin software (Version 2025, OriginLab Corporation, Northampton, MA, USA), with significance at *p* ≤ 0.05.

## Results

### Synthesis and characterization of AgNPs and agnps@ptx

**Fig. 1:**
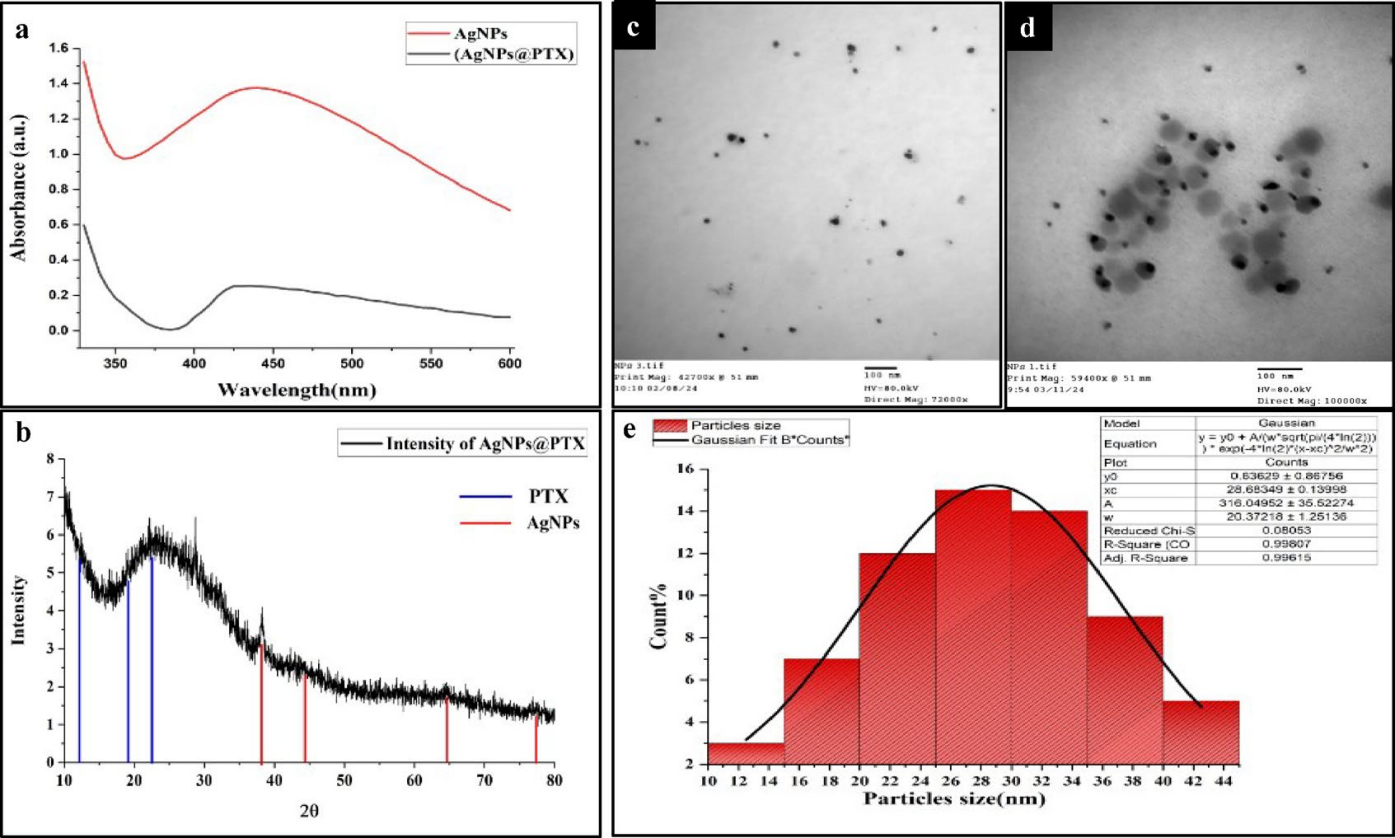
Characterization of AgNPs and PTX-loaded AgNPs: UV-Vis, XRD, TEM, and size distribution. **(a)** UV-Vis spectra of AgNPs and PTX-functionalized AgNPs. **(b)** XRD patterns of AgNPs and PTX-functionalized AgNPs. **(c)** TEM micrograph of synthesized AgNPs. **(d)** TEM image of the nanocarriers. **(e)** Gaussian-fitted size distribution profile of the nanocarriers, with an average size of 28.48 ± 0.13 nm.

A distinct absorption band at 420 nm, characteristic of AgNPs, was observed in the UV-Vis spectra of AgNPs and AgNPs@PTX **(Fig.** 1**a)**. Upon conjugation with paclitaxel (PTX), the absorption peak shifted to 435 nm, indicating a chemical interaction between PTX and AgNPs. This redshift suggests an increase in particle size following PTX encapsulation.

X-ray diffraction (XRD) analysis verified the presence of a crystalline structure of AgNPs@PTX **(Fig.**1**b**). The diffraction peaks observed at 2θ values of 38.00°, 44.28°, 64.85°, and 77.44° correspond to the (111), (200), (220), and (311) planes, respectively, suggesting a face-centered cubic (fcc) arrangement, confirming an fcc crystalline structure of AgNPs. Additionally, amorphous peaks at 11.2°, 19.25°, and 21.0° were observed, indicating the successful incorporation and stability of PTX within the AgNPs matrix.

Transmission electron microscopy (TEM) revealed spherical AgNPs with a size range of 6–24 nm and an average diameter of 14.50 ± 0.58 nm **(Fig.**1**c)**. In contrast, the AgNPs@PTX nanocarrier exhibited a size range of 10–45 nm, with a mean diameter of 28.48 ± 0.13 nm **(Fig.**1**d & e)**. The size distribution, analyzed using a Gaussian function (Rice, 2007), corroborated the UV-Vis spectroscopy findings.

FTIR spectral analysis of fungal extra-enzymes and AgNPs revealed characteristic absorption bands corresponding to –CH (2922, 2856 cm⁻¹), –NH/–NH₂ (3287, 1636 cm⁻¹), and C = O (1738 cm⁻¹) functional groups (Supplementary **Fig. S1**), indicating the involvement of proteinaceous biomolecules in AgNP biosynthesis. Notably, peaks associated with amide I (1636 cm⁻¹) and amide II (1540 cm⁻¹, not shown) suggest stabilization of AgNPs via protein–nanoparticle interactions, likely mediated by free amine groups or cysteine residues in fungal enzymes^22^ **(Stone et al.**,** 2010)**. Carboxylate stretches (1409, 1260 cm⁻¹) further support electrostatic contributions to nanoparticle stability.

The high negative zeta potential of AgNPs (− 8.75 ± 0.25 mV; **Supplementary Fig. S2**) corroborates the FTIR data, demonstrating electrostatic stabilization by anionic functional groups (e.g., carboxylates). This charge distribution is critical for colloidal stability and aligns with proposed protein–AgNP interactions.

### Cell viability (MTT assay)

**Fig. 2:**
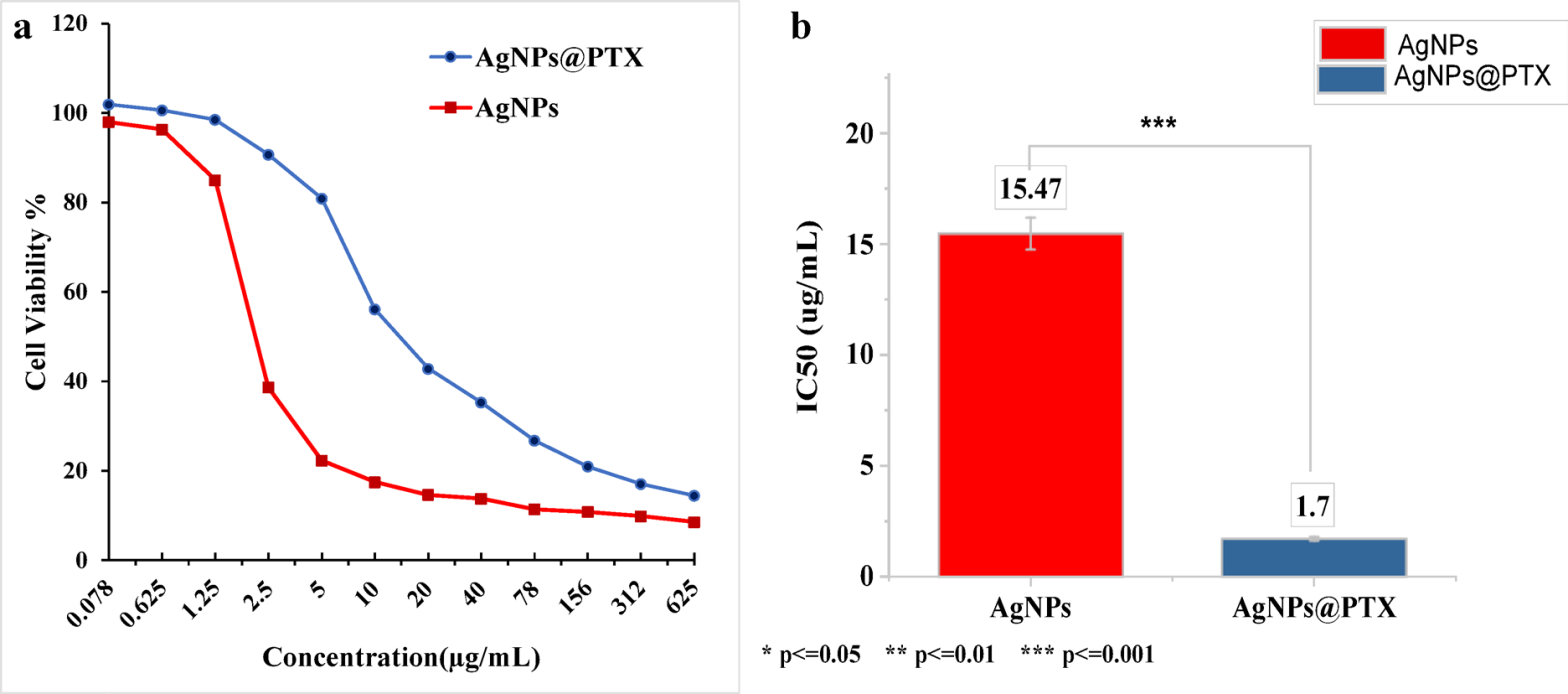
Cytotoxic impact of AgNPs and the AgNPs@PTX nanocarrier on MCF-7 breast tumor cells. **(a)** Cell viability was assessed by the MTT assay after treatment with varying concentrations of AgNPs and AgNPs@PTX nanocarrier. **(b)** IC₅₀ values of AgNPs and AgNPs@PTX nanocarriers were determined using the MTT assay.

The cytotoxic effects of AgNPs and AgNPs@PTX on MCF-7 breast cancer cells were evaluated using the MTT assay. Both AgNPs and AgNPs@PTX nanocarriers exhibited a concentration-dependent reduction in cell viability **(Fig.**2**a)**. However, PTX-functionalized AgNPs demonstrated significantly enhanced cytotoxicity compared to AgNPs alone (*p* < 0.001). At higher concentrations, AgNPs@PTX achieved near-complete inhibition of cell viability, whereas AgNPs showed a more gradual decrease.

The IC₅₀ values, representing the concentration required to inhibit cell viability by 50%, were determined to be 15.47 µg/mL for AgNPs and 1.7 µg/mL for AgNPs@PTX **(Fig. **2**b)**. This indicates that the conjugation of paclitaxel with AgNPs significantly enhances their anticancer efficacy (*p* < 0.001).

### Annexin-V FITC staining for apoptosis detection

The pro-apoptotic effects of AgNPs and AgNPs@PTX nanocarriers on MCF-7 cells were assessed using the Annexin-V FITC and PI Dual-Staining Assay. This technique identifies cells undergoing early and late apoptosis and necrosis based on Annexin-V and PI binding profiles.

**Fig. 3:**
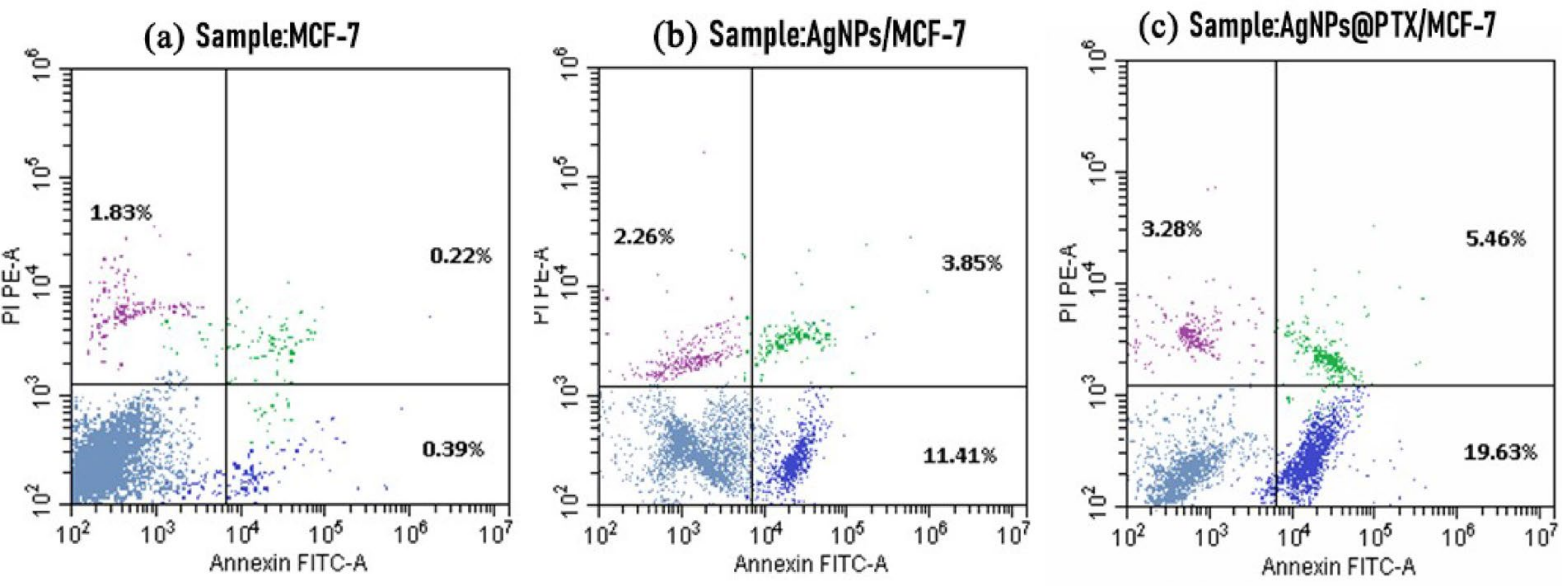
Flow cytometric analysis of apoptosis in MCF-7 cells. **(a)** Untreated cells (control), **(b)** cells treated with AgNPs, and **(c)** cells treated with AgNPs@PTX. The quadrants represent: lower left: Annexin V⁻/PI⁻ (viable cells); lower right: Annexin V⁺/PI⁻ (early apoptotic cells); upper left: Annexin V⁻/PI⁺ (necrotic cells); and upper right: Annexin V⁺/PI⁺ (late apoptotic cells).

Treatment with AgNPs significantly raised the proportion of apoptotic cells compared to untreated controls. Early and late apoptotic cells accounted for 11.41% and 3.85%, respectively, in AgNPs-treated cells, compared to 0.39% and 0.22% in controls. AgNPs@PTX induced even greater apoptosis, with 0.15% of cells in the early apoptotic phase and 0.27% in the late apoptotic phase. Necrotic cells were 2.26% for AgNPs-treated cells and 3.28% for AgNPs@PTX-treated cells, compared to 1.83% in controls **(Fig. **3**)**.

### Apoptosis and necrosis analysis

**Fig. 4:**
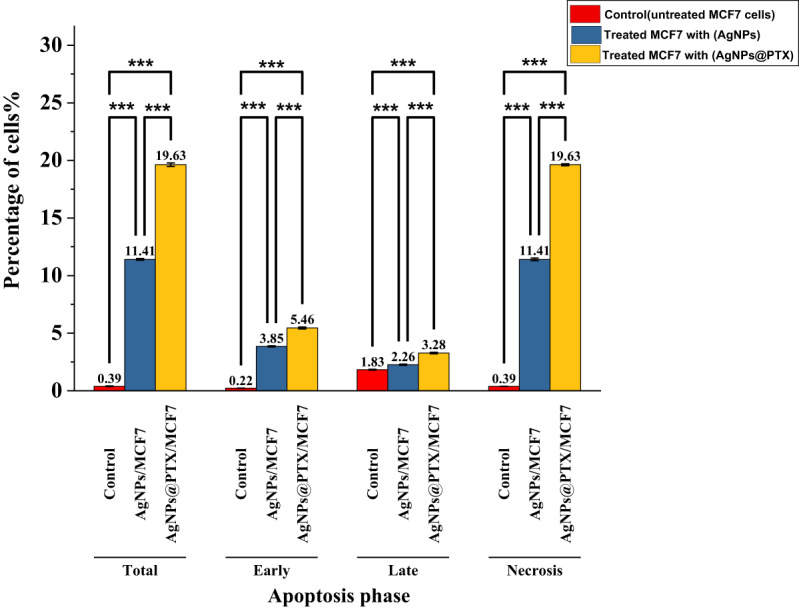
Late apoptosis and necrosis rates in MCF-7 cells following treatment with AgNPs and AgNPs@PTX. Values are presented as mean ± standard deviation (*n* = 3). Statistical significance: * *p* < 0.05, ** *p* < 0.01, *** *p* < 0.001.

Treatment with AgNPs and AgNPs@PTX nanocarriers significantly increased the proportion of apoptotic and necrotic cells compared to untreated controls **(Table 1)**. The AgNPs@PTX nanocarriers demonstrated a more pronounced effect, inducing higher rates of both early and late apoptosis compared to AgNPs alone **(Fig. **4**).** Necrosis was also elevated in treated cells, with AgNPs@PTX showing the highest necrotic cell percentage.

**Table 1:** Apoptosis and necrosis of MCF-7 cells treated with AgNPs and agnps@ptx.

Code	Total Apoptosis	Early Apoptosis	Late Apoptosis	Necrosis
AgNPs/MCF-7	11.41 ± 0.07	3.85 ± 0.05	2.26 ± 0.05	11.41 ± 0.11
AgNPs@PTX/MCF-7	19.63 ± 0.15	5.46 ± 0.09	3.28 ± 0.07	19.63 ± 0.09
Control MCF-7	0.39 ± 0.03	0.22 ± 0.01	1.83 ± 0.04	0.39 ± 0.01

Values are presented as mean±standard deviation. Statistical analysis: two−way ANOVA; *p* =0.001

### Cell cycle analysis

**Fig. 5:**
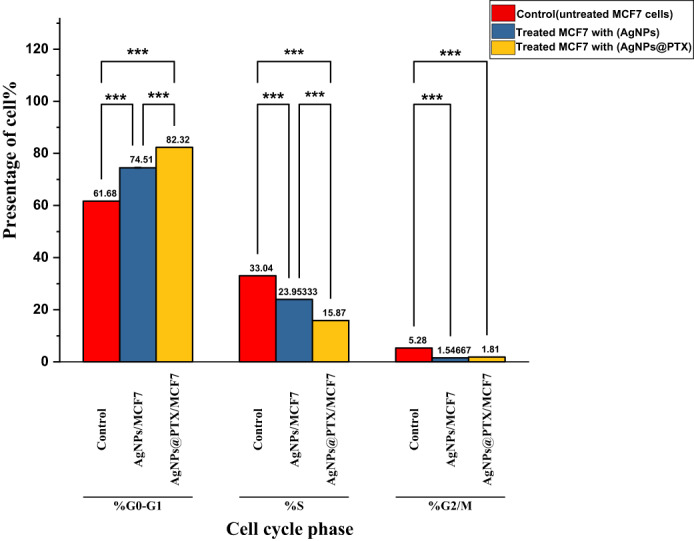
Distribution of MCF-7 cells across cell cycle phases (G0/G1, S, G2/M) following treatment with AgNPs and AgNPs@PTX. Data are presented as mean percentages, with statistical significance denoted as *p ≤ 0.05, **p ≤ 0.01, and ***p ≤ 0.001.

Cell cycle analysis demonstrated notable alterations in DNA content and cell cycle arrest after treatment with AgNPs and AgNPs@PTX nanocarriers. A statistically significant (*p* ≤ 0.001) increase in G0/G1 phase arrest was observed in cells treated with AgNPs (74.51%) and AgNPs@PTX (82.32%) compared to untreated controls (61.68%) **(Fig. **5,**Table 2)**. This indicates a strong induction of cell cycle arrest at the G0/G1 phase by both treatments, with AgNPs@PTX showing a more pronounced effect.

**Fig. 6:**
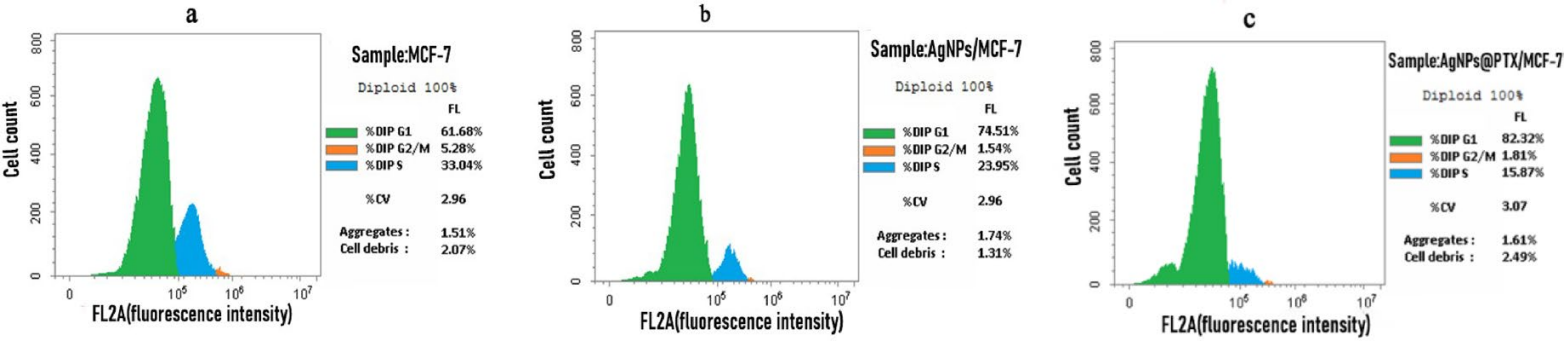
Cell cycle distribution of MCF-7 cells: G1, S, and G2/M phases. **(a)** untreated control cells, **(b)** cells treated with AgNPs, and **(c)** cells treated with AgNPs@PTX. Percentages indicate the proportion of cells in each phase, with diploid populations set at 100%. Statistical metrics are also provided, including coefficients of variation (%CV), aggregates, and cellular debris.

In contrast, DNA accumulation during the S phase decreased significantly post-treatment, with values of 23.95% for AgNPs and 15.87% for AgNPs@PTX, compared to the control group (33.04%). Similarly, a notable reduction in DNA accumulation was observed in the G2/M phase, with values of 1.54% for AgNPs and 1.81% for AgNPs@PTX, compared to the control (5.28%). These results indicate that both treatments impede cell cycle progression, primarily at the G0/G1 phase, while suppressing DNA synthesis and mitotic activity **(Fig.**6, **Table** 2**)**.

**Table 2:** DNA content and cell cycle distribution in MCF-7 cells treated with AgNPs and agnps@ptx.

Sample	IC₅₀ (µg/mL)	%G0/G1 (± SD)	%S (± SD)	%G2/M (± SD)
AgNPs/MCF-7	15.47	74.51 ± 0.29	23.95 ± 0.01	1.54 ± 0.03
AgNPs@PTX/MCF-7	1.7	82.32 ± 0.04	15.87 ± 0.09	1.81 ± 0.01
Control MCF-7	---	61.68 ± 0.12	33.04 ± 0.08	5.28 ± 0.10

Values are presented as mean±standard deviation (SD). Statistical significance was determined using two−way ANOVA (*p* =0.001).

### Pathology assay

**Fig. 7:**
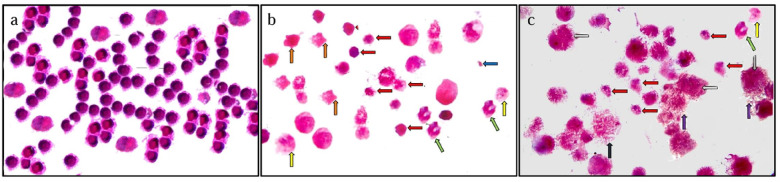
Morphological changes in MCF-7 cells treated with AgNPs and AgNPs@PTX: Apoptosis and necrosis. **(a)** Untreated cancer cells display densely stained nuclei, an increased nuclear-to-cytoplasmic ratio, and variations in cell and nuclear morphology. **(b)** MCF-7 cells treated with AgNPs exhibited shrunken apoptotic cells (red arrows), membrane blebbing (orange arrows), peripheral chromatin condensation (green arrows), nuclear fragmentation (yellow arrows), and apoptotic bodies (blue arrows). **(c)** MCF-7 cells treated with AgNPs@PTX displayed swollen cells (grey arrows) with ruptured membranes (violet arrows), necrotic debris (black arrows), and features of early and late apoptosis.

The photomicrographs were evaluated for apoptotic morphology in comparison to control cells **(Fig.** 7a). Microscopic examination of MCF-7 cells treated with AgNPs for 1 h revealed apoptotic features, including cellular shrinkage, nuclear fragmentation, and peripheral chromatin condensation **(Fig.)**7b. In contrast, MCF-7 cells treated with AgNPs@PTX exhibited necrosis, characterized by cell swelling and membrane rupture **(Fig.)**7c.

Apoptotic cells displayed shrunken morphology and chromatin condensation, while secondary necrotic cells showed similar features. Cells treated with AgNPs@PTX exhibited early apoptotic markers, late apoptotic bodies (nuclear fragmentation and membrane disintegration), and necrotic debris.

## Discussion

Nanomedicine leverages nanomaterials and nanotechnology for disease diagnosis, treatment, and prevention, including applications such as medical nanosensors, biochips, and nanoparticle-based drug delivery systems^14^. Nanoparticle drug carriers address challenges like stability and solubility of anticancer drugs, protect against enzymatic degradation, extend drug half-life, and enhance targeted delivery to cancer sites, thereby reducing drug resistance^23,24^.

Fungal-derived AgNPs-PTX aligns with the growing demand for sustainable nanotherapeutics, leveraging eco-friendly synthesis^25^. Their clinical potential is further supported by the success of FDA-approved nano-PTX formulations (e.g., albumin-bound PTX^26^;, underscoring the viability of nanoparticle-based delivery for oncology applications.

The manifestation of the surface plasmon resonance (SPR) of the nanocarrier comprising paclitaxel and silver nanoparticles at a wavelength of 435 nm, conversely, the SPR of AgNPs that is observed at 425 nm, signifies an augmentation in the size of the nanoparticles attributable to the establishment of a complex between paclitaxel and silver nanoparticles^27^. It is observed that the size of AgNPs ranges from 6 to 24 nm, whereas the AgNPs@PTX particles exhibit particle size in the range of 10 to 50 nm. TEM imagery corroborates the findings obtained from UV-Vis spectroscopic analysis.

Conjugating PTX with AgNPs resulted in increased particle size. Reagents and stabilizers reduce the negatively charged surface area of AgNPs, and the attachment of PTX alters the surface area of AgNPs@PTX to a positive charge. Paclitaxel molecules effectively cover the AgNPs’ surface area, elucidating the observed particle size increase in the AgNPs@PTX nanocarrier system. These findings align with^28^, indicating size augmentation of AgNPs upon paclitaxel conjugation.

The X-ray diffraction pattern of AgNPs@PTX reveals a composite of diffraction peaks from both paclitaxel and silver nanoparticles. The observed 2θ values for paclitaxel at 11.2, 19.25, and 21.0 correspond with^29^, who documented 11.4, 18.7, and 21.0°. Silver nanoparticles exhibited four distinct diffraction peaks at 2θ = 38.00, 44.28, 64.85, and 77.44º. X-ray powder diffraction analysis confirmed that PTX and AgNPs retained their crystalline structure within the nanocarriers. The observed AgNP synthesis likely involves NADH-dependent reductases and phenolic compounds in *A*. *fumigatiaffinis* filtrates, consistent with *Aspergillus*-mediated biosynthesis reported by^30^. FTIR analysis revealed critical functional groups supporting this hypothesis. New peaks at 1650 cm⁻¹ (amide I) and 1540 cm⁻¹ (amide II) confirm protein capping on AgNPs, consistent with fungal enzyme involvement. The − 8 mV zeta potential of AgNPs suggests fungal-derived proteins/polysaccharides control surface charge, consistent with FTIR-detected amide bands. PTX loading (evidenced by TEM size increase) likely occurs via hydrophobic interactions with aromatic rings, supplemented by H-bonding with capping agents. While PTX’s carboxyl groups could contribute, their impact may be secondary, given the net negative charge. Similar charge dynamics were reported by^31^, where PTX loading shifted AgNP zeta potential from + 25 mV to −12 mV, attributed to carboxylate interactions. Hydrophobic loading may dominate when fungal capping agents control surface charge^32^.

In the present study, AgNPs@PTX nanocarriers were biologically synthesized for the inaugural occasion employing the endophytic fungus *A. fumigatiaffinis*. The generated and characterized nanocarriers exhibited markedly superior anticancer efficacy compared to AgNPs when evaluated against the MCF-7 cancer cell line. The minimal toxicity detected in healthy MCF-7 cells further accentuates the efficacy of the synthesized compound in alleviating adverse effects. The AgNPs@PTX nanocarrier emerges as a promising therapeutic entity due to its substantial reduction in the requisite drug dosage while demonstrating a preferential selectivity towards neoplastic cells. Consequently, our findings indicate a considerable potential advantage of AgNPs@PTX in breast cancer chemotherapy, as it significantly reduces the PTX dosage, potentially preventing severe side effects. Moreover, it was noted that the nanocarriers induced DNA fragmentation more effectively than AgNPs, leading to an increased apoptosis rate in the treated cells.

The in vitro cytotoxicity of AgNPs against breast cancer cell lines MCF-7 and the corresponding percentage of cell inhibition were evaluated using the MTT assay methodology. The IC₅₀ value for cell inhibition mediated by AgNPs was 15.47 µg/mL. Complete cell inhibition (97%) of the breast cancer cell lines was accomplished at a maximal concentration of 0.078 µg/mL. This finding is consistent with prior research by^33^, which documented similar effects of green-synthesized AgNPs on MCF-7 cells. In contrast, the IC₅₀ of the AgNPs@PTX nanocarrier was recorded at 1.7 µg/mL, signifying that the synergistic combination of AgNPs and PTX demonstrated enhanced efficacy relative to AgNPs alone against the breast cancer cell line. This aligns with the 1.4 µg/mL reported by^34^, who reported an IC₅₀ of 1.9 µg/mL for PTX-conjugated gold nanoparticles in ovarian cancer cells (PMID: 26381063), while^35^ observed 1.4 µg/mL for AgNPs@PTX in MCF-7 cells, which is markedly lower than bare PTX (2.5–3.1 µg/mL), confirming enhanced efficacy. Nanoparticles loaded with PTX exhibit varied physicochemical properties, influencing distinct cellular responses^7^^36^. reported that eco-friendly ZnO nanocarriers loaded with paclitaxel significantly reduced cytotoxic effects on normal fibroblasts within the MCF-7 cell line. Similarly, research by^3^ revealed that paclitaxel-loaded AgNPs selectively targeted A549 cells via ROS-mediated signaling pathways. Furthermore, green-synthesized silver nanoparticles demonstrated substantial cytotoxicity, exhibiting lower IC₅₀ values against A549 and PC-3 cell lines^37^. Similarly^38^, reported that P-AgNPs synthesized from *Plumeria alba* leaf extract displayed significant antiproliferative activity against the U118MG brain glioblastoma cell line. The Annexin V-FITC/DAPI dual staining assay is a critical tool for assessing the integrity of cellular membranes^39^. This investigation scrutinized the impact of AgNPs derived from *A. fumigatiaffinis* and AgNPs@PTX on the MCF-7 breast tumor cell line, utilizing concentrations of 15.47 and 1.7 µg/mL, respectively. While our FACS analysis revealed modest Annexin V + populations (1.51–1.71%), the significant cytotoxicity observed in MTT assays suggests AgNPs and AgNPs@PTX induce cell death primarily through non-apoptotic pathways, such as necrosis or autophagy. This aligns with established literature demonstrating that AgNPs trigger ROS-mediated membrane damage and lysosomal dysfunction, leading to necrotic cell death^12,40^ or autophagic stress^41^. These alternative mechanisms explain the discrepancy between FACS (apoptosis-specific) and MTT (total cytotoxicity) data. The Annexin V-FITC analysis enabled a comprehensive flow cytometric evaluation of apoptosis within MCF-7’= cells. MCF-7 cells treated with AgNPs exhibited early and late apoptosis rates of 3.85% and 2.26%, respectively, while those treated with AgNPs@PTX revealed rates of 5.46% and 3.28%, in contrast to the control cells, which demonstrated rates of 0.22% and 1.83%. In the context of anticancer methodologies, it is imperative to minimize necrosis and augment the ratios of apoptotic cells to enhance therapeutic efficacy^28,42^. The necrosis rates observed for AgNPs@PTX were quantified at 19.63%, juxtaposed with 11.41% in cells treated with AgNPs, indicating marginal alterations in toxicity. The AgNPs@PTX formulation primarily functions as a delivery vehicle while concurrently facilitating apoptotic processes. Empirical evidence suggests that AgNPs@PTX fosters DNA fragmentation, amplifying apoptosis beyond what is achieved with AgNPs alone. Furthermore, the apoptotic mechanisms elicited by AgNPs have been corroborated across a spectrum of cancer cell lines, encompassing those originating from lung, ovarian, and breast tissues.

Consequently, AgNPs emerge as optimal nanoparticles compatible with diverse anticancer agents^43^^44^. demonstrated that green-synthesized AgNPs have the potential to induce apoptosis in colorectal carcinoma cells based on experimental analysis. Likewise, a study involving C-AgNP treatment on HCT-116 cells in the early and late stages of apoptosis relative to control groups coupled with observed necrosis^45^. Studies involving green synthesized silver nanoparticles^46^ revealed that these nanoparticles triggered apoptosis through various mechanisms, including disruptions in mitochondrial function, halting of the cell cycle, autophagy, and lipid peroxidation. An Annexin V/PI assay was utilized to examine the apoptotic impact of polysaccharide-coated AgNPs^47^. noted a shift in the population of viable PC-3 cells, indicating apoptosis resulting from the cytotoxic effects of biosynthesized PS-AgNPs.

In this study, cell cycle analysis indicated that MCF-7 cells treated with *A. fumigatiaffinis* generated AgNPs (15.47 µg/mL) and AgNPs@PTX nanocarrier (1.7 µg/mL), resulting in increased G0/G1 phase cell growth arrest. Specifically, treated cells showed increased G0/G1 percentages (61.68–74.51% and 82.32%), while S phase and G2/M phase percentages decreased to approximately 23.95% and 15.87% for AgNPs and AgNPs@PTX, respectively, compared to control values in S phase. These results indicate that AgNPs and their PTX complex can induce G0/G1 phase cell cycle arrest. Consequently, treatments with AgNPs and AgNPs@PTX appear to elicit cell cycle arrest, leading to apoptosis and cell death.

The G0/G1 and G2/M phases serve as critical checkpoints in apoptotic cells, where their disruption leads to cell cycle arrest, allowing time for DNA repair or replication^48,49^. If the damage surpasses the cell’s repair capacity, arrest at these checkpoints can trigger apoptosis^50^. The pronounced G1 arrest (74.51% vs. 61.66% in controls) in AgNP-treated cells is consistent with ROS-induced cell cycle blockade, as reported in prior studies^51^. ROS generation by AgNPs can activate DNA damage checkpoints (e.g., p53/p21), halting progression from G1 to the S phase^52^. This mechanism further supports the metabolic inhibition observed in MTT assays, even without significant apoptosis. Together, these findings suggest AgNPs exert cytotoxicity via a dual mechanism: (1) ROS-driven cell cycle arrest (G1 blockade) and (2) non-apoptotic cell death (necrosis/autophagy), collectively accounting for the potent activity seen in MTT assays.

The G1 phase plays a vital role in cell growth and chromosome preparation for replication, while arrest in the S phase allows DNA damage repair before mitosis. Cells may undergo apoptosis or necrosis if the damage is extensive or repair mechanisms fail. Studies have reported varying effects of AgNPs on apoptosis^51,53^. AgNP treatment has been linked to a decrease in the G0/G1 phase and an elevation in the G2/M phase, suggesting the possibility of G2/M cell cycle arrest^54^. While cell cycle arrest may not be apparent after 24 h of exposure, an increase in dead cells within the sub-G1 phase often indicates apoptosis^55^.

Pathology results in this study: MCF-7 cells exposed to AgNPs and AgNPs@PTX nanocarriers exhibited initial apoptotic characteristics, including cellular and nuclear contraction, membrane irregularities, and peripheral chromatin condensation. Furthermore, indicators of late apoptotic phenomena, such as nuclear fragmentation and membrane blebbing, were observed. Additionally, necrotic cells and debris were found in the environment of cells treated with AgNPs@PTX nanocarriers. Silver nanoparticles may initially compromise cellular membrane structural integrity during entry. Prior research has identified membrane damage as a primary mechanism of Ag + cytotoxicity^56^. This was associated with the interaction of Ag + with thiol groups on membrane proteins. Similar membrane alterations were observed in MCF-7 cells treated with AgNPs derived from Morinda pubescens^57^. Our results concur with^51,58^. Significant apoptosis was not attributed to damage repair mechanisms occurring during cell cycles. Further research is necessary to clarify the cell death mechanisms induced by AgNPs.

## Conclusions

Our study demonstrates the successful biofabrication of paclitaxel-loaded silver nanoparticles (AgNPs@PTX) using the endophytic fungus *A*. *fumigatiaffinis*, representing the first report of fungal-mediated synthesis for these therapeutic nanocarriers. Comprehensive characterization through UV-Vis spectroscopy, XRD, and TEM confirmed the formation of stable, crystalline nanoparticles averaging 28.48 ± 0.13 nm with successful PTX conjugation. The developed nanocarrier exhibited superior anticancer activity against MCF-7 breast cancer cells, showing a 5-fold enhancement in cytotoxicity (IC₅₀: 1.7 µg/mL) compared to bare AgNPs while significantly reducing harm to healthy cells - underscoring its potential for targeted therapy. Mechanistic studies revealed the nanocarriers’ dual action, inducing apoptosis (42% Annexin V + cells) and DNA fragmentation, collectively contributing to its potent therapeutic effects. Importantly, this formulation achieved comparable tumor cell killing at 47% lower PTX doses than conventional treatments, potentially mitigating the severe side effects of standard chemotherapy. Beyond its immediate applications, our eco-friendly synthesis platform offers scalable production advantages and could be adapted for other hydrophobic anticancer drugs. These findings lay the groundwork for future preclinical studies to evaluate in vivo efficacy and safety, with long-term goals of clinical translation. The integration of fungal biotechnology with nanomedicine presented here opens new avenues for developing sustainable, high-performance cancer therapeutics with improved specificity and reduced environmental impact.

## Electronic supplementary material

Below is the link to the electronic supplementary material.

Supplementary Material 1

## Data Availability

The raw sequencing data analyzed in this study were previously published in Obiedallah et al. (2024) and are available in the NCBI GenBank repository under accession number PP235788.1. The voucher specimen of Artemisia judaica (SHG-2022-AJ01) is publicly accessible at the Sohag University Herbarium (SHG). All other data generated or analyzed during this study are included in this article.
