# Supplementary material for: Exploring the anticancer potential of green silver Nanoparticles–Paclitaxel nanocarrier on MCF-7 breast Cancer cells: an in vitro approach
Source: Sci Rep. 2025 Jun 20;15:20198. doi: 10.1038/s41598-025-06275-4 (PMC12181376; doi:10.1038/s41598-025-06275-4)
Supplement: Supplementary file 1 — Supplementary Material 1 [file 41598_2025_6275_MOESM1_ESM.docx]

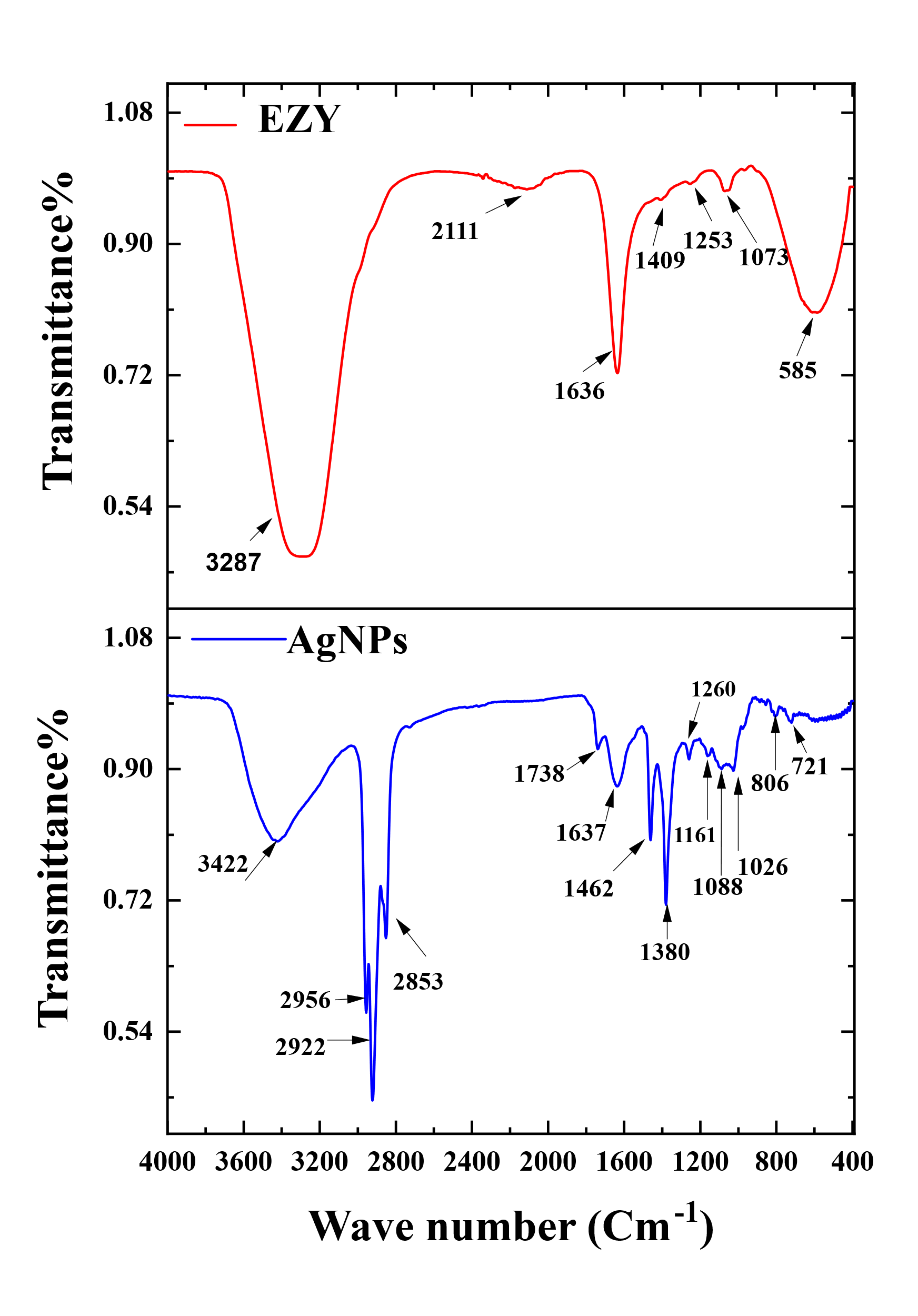


**Supplementary Fig. S1** FTIR spectra of **(a)** fungal extra-enzymes (EZY) and **(b)** biosynthesized silver nanoparticles (AgNPs). Attenuated total reflection (ATR)-FTIR spectra showing functional groups involved in AgNP biosynthesis and stabilization.


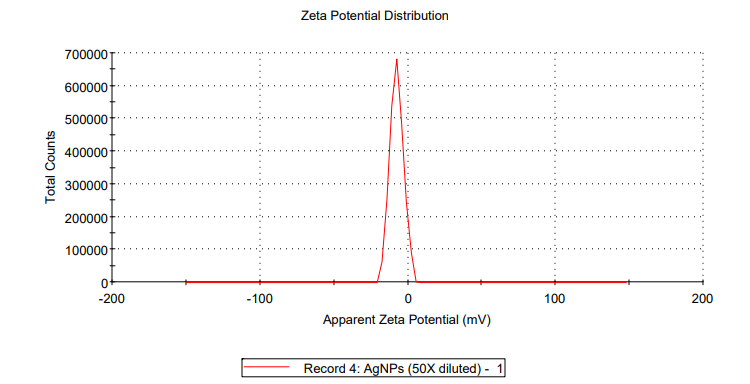


**Supplementary Figure S2** Zeta potential distribution of biosynthesized silver nanoparticles (AgNPs). Zeta potential histogram of AgNPs (50× diluted in deionized water) measured via electrophoretic light scattering.
